# Peer Review of “Machine Learning for Risk Group Identification and User Data Collection in a Herpes Simplex Virus Patient Registry: Algorithm Development and Validation Study”

**DOI:** 10.2196/28922

**Published:** 2021-06-11

**Authors:** José Alberto Benítez Andrades

**Affiliations:** 1 Department of Electric, Systems and Automatics Engineering School of Industrial, Computation and Aerospace Engineering Universidad de León León Spain

**Keywords:** data collection, herpes simplex, registry, machine learning, risk assessment, artificial intelligence, predictor, risk

*This is a peer-review report submitted for the paper “Machine Learning for Risk Group Identification and User Data Collection in a Herpes Simplex Virus Patient Registry: Algorithm Development and Validation Study.”*

## Round 1 Review

### General Comments

The authors of this research [1] discuss a platform containing a random forest classifier applied to the medical reports of patients suffering from the herpes virus. The manuscript describes an introduction to the proposed topic, the problem the authors intend to solve, the solution, and a discussion. Although the research seems interesting, the manuscript has some weaknesses that the authors must resolve.

### Specific Comments

#### Major Comments

1. Authors should read the authors’ guidelines at https://www.jmir.org/content/author-instructions. I suggest that they adapt their manuscript to the templates offered by JMIR; the title does not match the format proposed by the journal, the appendices do not have a caption, the tables can go in the manuscript, etc.
2. In relation to the content of the manuscript, there is no exhaustive bibliographic review in which existing studies applied to a classification problem such as the one the authors present are mentioned. Because of this, the justification for the development they propose is quite weak and can be improved upon.
3. Authors indicate that they separated the data sets by *train_test_split*; however, there is no clear description of the content of these two data sets. It is not known whether the classes are balanced or not, and no data preprocessing was done to ensure that the generated model is optimal for any type of data. Authors should indicate if they have done a cross-validation when training their model or not. If not, I recommend that they do it.
4. It would be enlightening to show the matrix of confusion as well as to indicate in a table a comparison of the measures of precision and accuracy on random forest with different hyperparameters.
5. To search for the best hyperparameters, I suggest using GridSearchCV or similar.
6. Finally, it is necessary to make a comparison between the proposed model and others that already exist.
7. Authors are requested to upload their code and the models to a repository to guarantee their reproducibility.

## Round 2 Review

I thank the authors for their work in improving this manuscript. They have responded correctly to all my suggestions, and I consider that the manuscript has improved in quality and can be considered for publication in this journal.
